# If you digress, shall we not neglect? Investigating the moderating role of individual cultural values while predicting job neglect among doctors as footprint of psychological contract violation

**DOI:** 10.1186/s40359-025-03948-7

**Published:** 2026-01-22

**Authors:** Muhammad Bilal Ahmad, Fizza Rizvi, Nausheen Shakeel, Amna Niazi

**Affiliations:** 1 Hailey College of Banking & Finance, University of the Punjab, Lahore, Pakistan; 2 Division of Management & Administrative Sciences, University of Education, Lahore, Pakistan; 3 Humanities and Social Science Department, University of Engineering and Technology, Lahore, Pakistan

**Keywords:** Psychological contract violation, Job neglect, Mastery orientation, Subjugation orientation

## Abstract

**Background:**

Services provided by the health care sector is imperative for the society. Therefore, deviant behaviors among doctors is crucial to address for ensuring high-quality patient care, maintaining healthcare standards, and enhancing overall organizational efficiency in public sector hospitals. This study aims to predict deviant behavior resulting from psychological contract violations among doctors employed in public sector hospitals in Pakistan. It also examines the moderating effects of individual cultural differences, particularly mastery orientation and gender. The objective of the study is to emphasize on the factors contributing in negative behavioral outcomes in healthcare sector resultantly providing compromised services to the patients.

**Method:**

A cross-sectional, two-way research design was employed, targeting doctors working in public hospitals. Structured questionnaires were employed for data collection. Data analysis was conducted using IBM SPSS and Smart-PLS 4.0, employing partial least squares structural equation modeling.

**Results:**

The findings reveal a significant positive relationship between psychological contract violation and job neglect. Furthermore, mastery orientation was found to moderate this relationship: doctors with high mastery orientation were less likely to exhibit deviant behavior in response to psychological contract violations compared to those with low mastery orientation. Gender was also examined as a moderating variable with significant impact.

**Conclusion:**

The study highlights the importance of managing counterproductive behaviors among doctors by implementing appropriate pre- and post-intervention strategies. Limitations and directions for future research are also discussed.

## Introduction

The issue of negligence among healthcare professionals is a growing concern, particularly in public sector hospitals where resource constraints, high patient loads, and limited workforce availability often exacerbate the problem [1, 2]. Doctors, as key members of the healthcare workforce, are responsible for providing essential medical services. Any neglect in performing duties may result in severe consequences and can endanger stakeholders’ interests. However, in many public hospitals, job neglect behaviors have been observed, including tardiness, extended breaks, slower work pace, and reduced effort during working hours. Such behaviors can severely compromise the quality of patient care, leading to patient dissatisfaction and increased pressures on the healthcare system [3].

There is a drastic increase in the population of Pakistan and too many people need care, and due to lack of staff the doctors feel pressurized. Constant shortage of resources, excessive workload, unwanted delays in promotions, staff drowning in work, and equipment always just out of reach. All this ramps up the odds that doctors feel let down by their employers. In conclusion, when doctors start cutting corners, neglect their duties, it does not just hurt their reputation, but the patients can really suffer. In Pakistan, this issue is particularly pronounced, where public hospitals suffer from a lack of sufficient healthcare professionals, resulting in an overburdened workforce. As a consequence, doctors may engage in unproductive behaviors such as negligence, which negatively impacts patient care. Job neglect of such extents has repercussions on both the health of patients and the reputation and performance of the health care system as patients are forced into private sector treatment, placing additional strains on the publicly funded health care system.

The breach of the psychological contract between employers and doctors may contribute to these phenomena. Psychological contracts are the informal obligations and expectations between the [4] employee and employer, that when breached can result in negative affect and behavior [5]. In the specific context of PCV, employees may perceive betrayal with negative emotions or attitudes, which will in turn result in work disengagement, job dissatisfaction, and job neglect [6]. Social exchange theory, presented by Blau in 1964 explains this phenomenon in detail [7]. It is stated in the theory that the relationship between employer and the employee is based on a reciprocal relationship. If the employees believe that the organization is working for the betterment of people they also reciprocate but the moment the employees feel that the organization is not giving them the right treatment that they deserve they change their attitude accordingly. Restubog et al. [8] argued that negative acts of silencing have an emotional dimension and can also be expressed as cognitive and behavioral withdrawal resulting in an individual becoming less devoted to an organization and, consequently, lower productivity.

Clinically, the ability to provide the best of services, which is the hallmark of the healthcare system, makes it imperative to appreciate the psychological contract that exists between employees and employers to determine what may drive employees to actively refrain from work (or work avoidance). It is maintained in the literature that employees subjected to a psychological contract breach are likely to respond in what may be characterized as negative or retaliatory behaviors of which neglecting work is a form of passive retaliation [9]. The consequences of the breach are borne by the individual more so than they are by the organization in the healthcare setting. When a breach of contract occurs in the form of PCV, the consequences— as noted in the literature [10] in such a case as service employees are likely to disengage from work as a form of passive work avoidance countermeasure and, as a result, service quality lacking.

Other individual variances, such as cultural orientation and gender, also play a vital moderating role between psychological contract breach and job neglect behaviors. Scholars have documented cultural elements as an important factor how workers interpret and respond to contract breaches [8, 11]. The mastery orientation of the individual, that is the motivation to pursue goals and develop personally, may shape the level of their response to perceived contract breaches. For instance, high mastery-oriented doctors may not engage in neglectful behaviors, even while undergoing contract breaches psychologically, while those low in mastery orientation may exhibit more negative responses [12].

Considering the individual differences outlined earlier, the present study aims in predicting the negative, particularly neglectful, behaviors that stem from violated psychological contracts among public sector doctors in Pakistan. By exploring these moderators, the study hopes to inform policy makers and improve practices in health sector management to reduce neglectful behaviors and promote progress in the workplace atmosphere.

### Psychological contract violation and job neglect

The psychological contract captures the exchange-deal between organizations and employees. Mutual expectation and obligation stem from both parties [13]. It depends on employees’ subjective perceptions on what the employer ought to satisfy [14]. A psychological contract violation (PCV) happens when employees think that there has been a breach of these unspoken commitments, and denial, let downs, dismissal, and anger is felt when the organization’s promises begin to shatter [15–17].

Studies conducted by multiple authors [18, 19] have also treated job neglect as an organizational deviance construct linked with psychological contract violation. Job neglect is a behavioral manifestation of PCV, characterized by reduced effort, inattention, absenteeism, delayed tasks, personal distractions, and slow work pace [20]. It reflects disengagement and withdrawal, often prompted by work dissatisfaction [21]. Rai and Agarwal [22] noted that PCV undermines work engagement, leading to gradual disengagement. Topa et al. [23] further emphasized that the psychological contract robustly predicts both personal and organizational outcomes. The Conservation of Resources theory underscores that unmet obligations cause resource loss, psychological strain, lower job satisfaction, and increased neglect [24]. According to Rayton and Yalabik [25] perceived contract violations significantly erode job satisfaction, while Flickinger et al. [26] found that PCV diminishes confidence and boosts turnover intentions. In healthcare contexts, Collins [27] demonstrated that contract breaches lead to reduced satisfaction, well-being, and increased job neglect when expectations in training and career development are unmet.

Recent research continues to highlight the detrimental impacts of psychological contract breaches. For instance, He et al. [24] found that PCV is a positive predictor of turnover intention among knowledge workers. Shin and Shin [28] examined technostress in the tourism sector and determined that technostress amplifies psychological contract violations and, in turn, resistance to organizational change. Meanwhile, De Clercq [29] identified that psychological contract breach increases employees’ intentions to quit, with proactive personality acting as a moderating factor.

In this research, deviant behavior refers to organizational deviance, involving activities that breach organizational norms and impede the efficient function of public sector hospitals. While deviant behavior is a broad construct, our interest focuses on behaviors negatively influencing quality of service and the overall effectiveness of operational performance. Behaviors such as lateness, carelessness, rule violations, and job neglect fall clearly within the domain of organizational deviance. Deviant behaviors, including silent withdrawal, frequently result from PCV [30]. Affective Events Theory suggests negative psychological states explain declines in job satisfaction, trust, and well-being—but often manifest more in attitudes than performance [23, 31]. Kwonet al. [32] described how repeated violations trigger dysfunctional behavior, while Liang et al. [33] reported that PCV exacerbates frustration, inciting maladaptive responses. Si et al. [34] empirically confirmed a positive relationship between PCV and job neglect, including longer rests, reduced work quality, errors, and socializing at work.

When doctors feel like their organization has broken promises in form of piling on too much work, ignoring their efforts, blocking career growth, or just not having their backs they experience cognitive and emotional dissonance. They see it as unfair and even consider it a betrayal. This sense of injustice hits hard, which stirs up frustration, reduces motivation, and leaves the doctors to change their own values. As a reaction they may show up late, let work slide, cut corners, or skip steps they know matter. In hospitals, where the pressure never really lets up and everyone’s stretched thin, this kind of withdrawal stands out even more. It’s not just a bad mood—psychological contract violations set off a real shift from caring to coasting. Grounded in social exchange and resource conservation theories, it is argued that perceived violations of the psychological contract lead employees to respond with reduced effort and disengagement. Empirical evidence from research [24, 27–30], consistently demonstrates that PCV undermines employee engagement and increases counterproductive behaviors, such as job neglect.

Thus, the study posits the following hypothesis:

- H1: *Psychological contract violation (PCV) is positively related to job neglect.*

### Cultural values moderating effects

Culture has long been recognized as central to conceptualizing the psychological contract [35], yet most early research overlooked how cultural values shape contract formation and violation responses. Thomas et al. [36] argued for the importance of individual-level cultural variations, such as mastery and subjugation, in influencing psychological contract dynamics. Arshad [12] extended this by showing that these cultural orientations moderate the effect of psychological contract violation (PCV) on turnover intentions at the individual level, not just between nations [37].

Specifically, individuals high in subjugation orientation (SO), who perceive themselves as subject to external forces, tend to interpret unmet obligations differently. They may not view these as contract violations unless expectations are severely breached, due to a sense of external locus of control [12, 38, 39].

In contrast, those with strong mastery orientation (MO) believe in their ability to influence and manage the environment. Such individuals may interpret PCV as necessary for long-term organizational health and thus may not react adversely or as strongly to unmet commitments, viewing them instead as acceptable trade-offs [40].

Beyond individual-level culture, national cultural values also moderate PCV’s impact. Jayaweera et al. [41] found that while national cultural practices mediate the relationships between psychological contract breach (PCB) and outcomes like turnover or performance, uncertainty avoidance culture did not significantly moderate behavior outcomes. More recently, the influence of cultural context has been reinforced in studies linking power-distance cultural orientations and organizational justice perceptions to behavioral responses such as retaliation and violation coping strategies [42].

Taken together, these studies underscore that cultural value orientations, both individual and national, shape how employees perceive and respond to psychological contract violations. While early exchanges and trust are cognitively shaped, cultural lenses determine whether employees interpret unmet commitments as violations, how severely they react, and what behavior follows. By examining SO and MO, you harness the insight that individual internal cultural values play a crucial role in moderating adverse behavioral outcomes like job neglect.

### Subjugation Orientation (SO)

Subjugation Orientation (SO) is the belief that one is a subject of external control and cannot significantly affect what is around them [43]. Individuals with high SO, tend to see unfulfilled contracts in a psychological context as the result of external and situational factors. This implies the high SO individuals do not perceive an organization’s broken promises as a violation of the psychological contract. They generally tend to see unfilled obligations as the product of organizational processes and of exogenous factors such as resources.

This is a cognitive mechanism that helps to tamp down the feeling itself, reduce the likelihood that the workers will take the emotionally confused step of shrinking their duties at work. Studies have also underscored the relevance of personal characteristics in explaining business situations, as it has been shown that certain personality characteristics can moderate the impact of psychological contract breaches [31].

In accordance with the Social Exchange Theory (SET) [7], those high in SO are less likely to retaliate in a negative manner as they may not have recognized the violation as intentioned. They are likely to feel a lack of reciprocity in the unmet obligations. The Conservation of Resources Theory (COR) [44] argues that people with high SO do not see unsuccessful commitments as a loss of resources. When there is no loss of resources, the level of psychological strain remains at a low level so that no further actions, including work avoidance, are precipitated. The Affective Events Theory (AET) [45] also proposed that the emotional reaction to organizational events such as a PCV would affect subsequent behavior. More particularly, individuals with high SO would have less negative feelings to a PCV and thus probably much less tendency for any type of work avoidance response.

Recent research has explored personal differences in emotional reactions following work-related events and their impact on behavior [31]. In health care where SO is high, physicians might accept the limitations of the entity or the un-kept promises as systemic issues. This mindset limits their chances to withdraw or cut down their work demands even when they feel that the psychological contract has been breached. More specifically, it has been found that how practitioners perceive organizational support and fairness can also affect work attitudes and behaviors such as job neglect [46, 47].

- H2: *SO moderates the relationship between PCV and job neglect (JN) among doctors working in public healthcare hospitals, such that the relationship is weaker for individuals high in SO.*

### Mastery Orientation (MO)

An individual believes that he or she can control or influence his or her outer world is referred to as Mastery Orientation (MO) [43]. High-MO people are proactive hence they believe that they can modify or adjust aspects of their environment to best fit their needs. In the framework of PCV, people with high MO are more likely to construe unfulfilled obligations as being temporary reversals as opposed to violations of trust. They can see missed commitments as contributing to the overall dimensions of institutional and/or systematic changes that are required for successful long-term development. Individuals with such qualities view the organizational setback as a tolerable byproduct of the wider objectives/changes, which makes it easier to stay invested and persist. In addition, Conservation of Resources Theory (COR) [44] proposes that those high in MO view challenges as resource-generating or resource-creating opportunities. Instead of withdrawing effort following a breach, they are likely to invest more into their work to restore equilibrium. In addition, Affective Events Theory (AET) [45] posits that workplace events result in affective reactions that ultimately influence attitudes and behavior. Individuals with high-MO may be more likely to engage in emotional challenges with adaptive strategies, interpreting the violation as a problem to solve rather than a cue to disengage. A recent study has investigated how people’s goals might affect their responses to challenges in the workplace, including their reactions to psychological contract breaches [48, 49]. In health care, physicians with high MO may perceive any unmet obligations (i.e., lack of training or resources) as difficulties to be surmounted rather than transgressions. This viewpoint prevents PCV from detracting from job neglect, while they are pursuing the broader goal, enhancing patient care and organizational systems. Organizational support and fairness have been shown to be the antecedents of healthcare professionals’ attitudes, and behaviors at work such as job neglect [48].

- H3: *MO moderates the relationship between PCV and JN among doctors working in public healthcare hospitals, such that the negative impact of PCV on job neglect is attenuated for individuals high in MO.*

### Multigroup modelling, gender moderation

There is additionally a claim regarding the preceding combinations of individual culture, which are similar to the structures of personality, i.e., the locus of management [50]. The dispute emerged in such a way that it could distinguish two dissimilar directions. According to Hofstede [51], on a conceptual basis, there’s a big variation between personality and culture: culture is considered as an external variable that’s influenced by personal and human behavior [12, 52]. Environment is provided by the culture in which personality is developed [53]. Gender is a personal dimension.

In recent years, gender has received increasing attention in organizational behavior research, and especially in relationship to how people engage, when their psychological contracts are violated (PCV). According to Social Exchange Theory (SET) [7], individuals’ attitudes in relation to PCV are influenced by their perceptions of fairness in the exchange and in the ongoing exchange relationship. Since men and women may have different social roles, expectations, and coping mechanisms, these gender differences can describe the way violations are construed and consequently how they matter to work behaviors such as job neglect. Gendered expectations often influence the way individuals choose to construct meaning of organizational events and the coping strategies they adopt-and for men and women, this can be quite different.

Also, AET [54] supports the view that gender can influence emotional responses or effects of events within the workplace. It is supported by the view that women may be more inclined to use adaptive coping strategies-that include seeking social support and joint problem-solving to an immediate course of action such as job neglect or withdrawal.

It is at work that the difference will most likely surface on the role of moderating variables such as Mastery Orientation (MO) and Subjugation Orientation (SO), particularly on women’s moderation of the relationship between PCV and job neglect. This would be in tandem with existing literature that posits that gender nuances emotional responses and behavioral outcomes emanating from PCV [22, 23].

Hence, the study proposed:

- H4: *Gender moderates the relationship between psychological contract violation (PCV) and job neglect (JN), such that the effect of PCV on job neglect is stronger for males than for females, and the moderating effects of Subjugation Orientation (SO) and Mastery Orientation (MO) are stronger for females than for males.*

The theoretical model proposed in this study combines psychological contract violation, job neglect, mastery orientation, and gender into an integrated framework rooted in Social Exchange Theory, Equity Theory, and culturally embedded behavioral mechanisms. Psychological contract violation serves as the main antecedent, as it triggers negative cognitive appraisals and emotional reactions resulting from perceived imbalance and broken reciprocity. These reactions give rise to conditions that help the development of behavioral withdrawal, manifesting in job neglect as a form of organizational deviance. This shift may be explained by Social Exchange Theory as reduced reciprocal effort after employees feel the organization has failed to repay promised obligations. This mechanism is further underpinned by Equity Theory, which suggests that individuals try to reestablish fairness by adjusting their inputs, often by putting in reduced effort or behaving in a neglectful way. All these constructs combined form an integrated, cohesive, and theoretically logical theoretical model that substantiates the relationship through which psychological contract violations translate into deviant behaviors within the culturally specific context of Pakistan’s public sector hospitals.

**Fig. 1:**
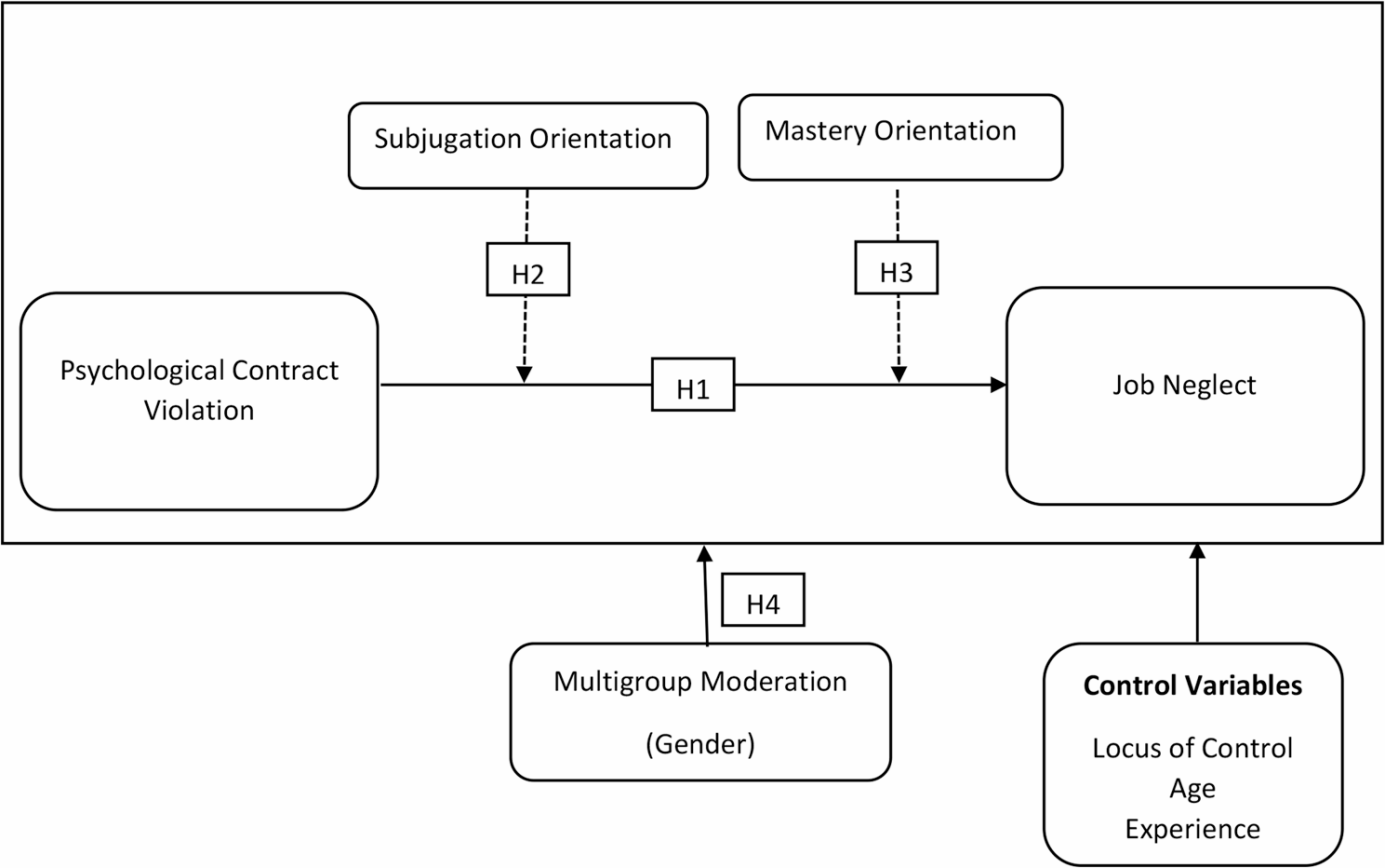
Theoretical framework (From Authors’ own source)

The study proposed the following theoretical model (See Fig. 1).

The figure represents the theoretical framework of the study, where Psychological Contract Violation effects the Job Neglect through the moderating role of Subjugation Orientation and Mastery Orientation, with multigrroup moderation of Gender.

### Research methodology

This study investigates negligence behaviors among doctors in public sector hospitals, using a quantitative, deductive, and cross-sectional two-wave design. To address the concern of common method variance [55], several measures were adopted in the study. The data were collected using convenience sampling from doctors working in tertiary care public hospitals in Lahore, Pakistan, at two times. Convenience sampling enabled the researcher to include participants who were easily accessible and agreeable to participate. Participants were contacted through institutional networks. They were given information about the study’s objectives, volunteered participation. In the first wave, 335 doctors were contacted. At time I, the data were collected on PCV (IV), JN (DV), fear of COVID-19 (marker variable), locus of control (control variable), and demographics from 265 respondents out of 335. In the second wave, conducted two weeks later, data on moderators (i.e., mastery orientation (MO) and subjugation orientation (SO)) were collected from 248 of the initial respondents who had already participated in the first wave of data collection. After initial screening for missing values and outliers, the final sample size was 240, yielding an overall response rate of 68.5%. On average, respondents were 30 years old, 57% were male, and 43% were female participants in the study. Most of the respondents were young and single (i.e., 90%) in the study. On average, most of the participants had less than 5 years of experience after starting a formal career in health care.

### Instruments and measures

A five-point Likert scale was used to measure the variables, with the scale ranging from 1 (Strongly disagree) to 5 (Strongly agree). Psychological contract violation was measured using the adapted scale of Robinson [56], which includes four items, with a composite reliability of 0.91 and a Cronbach’s alpha of 0.87. A sample item was “I feel extremely frustrated by how I have been treated by my organization”.

Job neglect was measured using a five-item scale developed by Rusbult [57], which has a composite reliability of 0.89 and a Cronbach’s alpha of 0.85. An example of the items in this measure is “I care very little about what happens to this hospital as long as I get a pay.” Mastery Orientation and Subjugation Orientation were measured using the Cultural Perspective Questionnaire 4 (CPQ4) which was developed and validated by Maznevski et al. [43], which has a Cronbach’s alpha of 0.84, a composite reliability of 0.88, and a Cronbach’s alpha value of 0.85, respectively. “Good performance comes from taking control of one’s work” and “It’s best to leave problem situations alone to see if they work out on their own” are the sample items of the measures of mastery orientation and subjugation orientation”. CPQ4 has been used in multiple psychological studies with other nationalities as well [12, 58].

#### Control variables

Locus of Control was measured using a four-item scale developed by Nießen 2022 [59]. Age and experience were included as control variables based on previous research by Vantilborgh and Bal [48, 60]. Data analysis was conducted using PLS-SEM with Smart PLS 2.0.

#### Common method bias (Marker Variable)

The Latent Marker Variable technique was used to counter the issue of common method variance Chin [61]. The Marker Variable, i.e., Fear of Covid, developed by Ahorsu [62, 63], with 07 items, was incorporated while collecting the data. It reduces the chance of CMV by 80% [61]. By running Harmon’s single-factor test, the problem of variation of the common method in data was tested. Results show that the test value is below the minimum level (i.e., < 0.50). Therefore, there is no problem of variation in the usual way it was presented in the data set. All basic assumptions for analysis of multivariate data were met for analysis.

## Results

SmartPLS 4 was used for the data analysis. Missing values and outliers were assessed before the data analysis. VIF values also showed no concern of multicollinearity. SEM was applied and the two-step approach was adopted. Measurement model first, followed by the structural model. For indirect paths, bootstrapping procedure with 5000 resamples was run.

In the measurement model, the confirmatory factor analysis model was used to test the extent to which the variable is distinguished. All the values regarding checking the measurement model reliability and validity are within the range of their threshold level. For convergent validity, AVE values for all the constructs are above the threshold level (i.e., > 0.5), and loadings of all items related to respective constructs are between threshold levels 0.6 and 0.8 [64]. The value of composite reliability for checking the internal consistency reliability was above 0.7 [65] for all constructs that support that there exists reliability in the data (See Table 1). Discriminant validity is tested in three ways: the Fornell and Larcker [66] method (the square root of AVE must be greater than other correlated values on other constructs) (See Table 2), by cross-loadings (See Table 3) of each item by respective constructs (all indicator loadings were higher than cross-loadings, which identified discrimination at the level of items) and through HTMT values (i.e. < 0.85) (See Table 4). All values fulfil the criteria mentioned to establish discriminant validity in the data.

**Table 1:** Measurement model

Latent Variable	Indicators	Loadings	Indicators Reliability	Composite Reliability	Cronbach’s alpha	AVE	Discriminant Validity
PCV	PCV1	0.80	0.64	0.91	0.879	0.732	Yes
	PCV2	0.89	0.80				
	PCV3	0.85	0.72				
	PCV4	0.88	0.78				
JN	JN1	0.68	0.46	0.89	0.852	0.58	Yes
	JN2	0.79	0.63				
	JN3	0.70	0.50				
	JN4	0.77	0.60				
	JN5	0.81	0.65				
	JN6	0.78	0.61				
MO	MO1	0.76	0.58	0.90	0.89	0.61	Yes
	MO2	0.83	0.70				
	MO3	0.82	0.68				
	MO4	0.80	0.63				
	MO5	0.74	0.55				
	MO6	0.73	0.53				
	MO7	0.79	0.63				
SO	SO1	0.68	0.46	0.887	0.853	0.53	Yes
	SO2	0.78	0.60				
	SO3	0.77	0.60				
	SO4	0.62	0.39				
	SO5	0.74	0.55				
	SO6	0.69	0.48				
	SO7	0.80	0.64				
ELC	ECL1	0.9	0.81	0.852	0.782	0.747	Yes
	ECL2	0.71	0.50				
ILC	ILC1	0.81	0.65	0.870	0.725	0.772	Yes
	ILC2	0.94	0.89				
Fear	Fear4	0.74	0.54	0.872	0.856	0.633	Yes
	Fear5	0.77	0.60				
	Fear6	0.72	0.52				
	Fear7	0.93	0.87				

**Table 2:** Cross loadings

	ELC	Fear	ILC	JN	MO	PCV	SO
ELC1	0.996	0.084	-0.057	0.130	-0.107	0.147	0.111
ELC2	0.709	0.122	-0.058	0.017	-0.019	0.061	0.006
Fear4	0.082	0.735	0.081	-0.048	0.174	-0.036	-0.109
Fear5	-0.007	0.774	0.011	-0.050	0.149	0.000	-0.092
Fear6	0.066	0.723	-0.022	-0.011	0.094	-0.056	-0.088
Fear7	0.107	0.932	-0.003	-0.147	0.164	-0.066	-0.135
ILC1	-0.030	0.029	0.806	-0.115	0.145	-0.173	-0.209
ILC2	-0.066	0.008	0.945	-0.207	0.255	-0.202	-0.194
JN1	0.018	-0.104	-0.103	0.680	-0.429	0.312	0.365
JN2	0.085	-0.138	-0.151	0.794	-0.458	0.489	0.484
JN3	0.067	-0.035	-0.066	0.704	-0.362	0.455	0.377
JN4	0.080	0.005	-0.165	0.774	-0.437	0.358	0.429
JN5	0.153	-0.185	-0.226	0.808	-0.577	0.409	0.479
JN6	0.129	-0.068	-0.150	0.783	-0.493	0.316	0.424
MO1	-0.079	0.198	0.234	-0.437	0.759	-0.383	-0.446
MO2	-0.173	0.141	0.305	-0.541	0.835	-0.427	-0.527
MO3	-0.163	0.092	0.223	-0.509	0.824	-0.467	-0.594
MO4	-0.081	0.259	0.160	-0.438	0.796	-0.393	-0.508
MO5	-0.012	0.036	0.134	-0.440	0.740	-0.315	-0.469
MO6	0.029	0.162	0.099	-0.455	0.725	-0.257	-0.416
MO7	-0.047	0.149	0.143	-0.508	0.792	-0.385	-0.503
PCV1	0.052	-0.057	-0.154	0.365	-0.381	0.797	0.448
PCV2	0.185	-0.030	-0.216	0.454	-0.443	0.894	0.511
PCV3	0.072	-0.068	-0.121	0.388	-0.356	0.848	0.416
PCV4	0.154	-0.040	-0.224	0.529	-0.458	0.881	0.552
SO1	0.054	-0.112	-0.203	0.359	-0.466	0.350	0.676
SO2	0.134	-0.121	-0.169	0.505	-0.537	0.320	0.777
SO3	0.045	-0.067	-0.199	0.453	-0.498	0.402	0.774
SO4	-0.008	-0.119	-0.084	0.294	-0.311	0.399	0.622
SO5	0.057	-0.095	-0.079	0.392	-0.430	0.514	0.743
SO6	0.087	-0.081	-0.077	0.285	-0.369	0.466	0.691
SO7	0.119	-0.120	-0.264	0.505	-0.551	0.496	0.798

**Table 3:** Fornell-Larcker criterion

	Median	Skewness	Kurtosis	VIF	ELC	Fear	ILC	JN	MO	PCV	SO
ELC	-0.067	-0.837	0.358	1.035	0.865						
Fear	0.385	-1.261	2.346	1.054	0.092	0.795					
ILC	0.318	-0.615	-0.314	1.080	-0.059	0.017	0.878				
JN	-0.310	0.181	-1.004		0.121	-0.121	-0.195	0.759			
MO	0.308	-0.479	-0.932	1.799	-0.101	0.187	0.241	-0.611	0.782		
PCV	0.014	-0.043	-1.113	1.563	0.142	-0.055	-0.214	0.516	-0.483	0.856	
SO	-0.106	0.019	-1.057	1.982	0.103	-0.140	-0.223	0.566	-0.635	0.569	0.728

**Table 4:** HTMT values

	ELC	Fear	ILC	JN	MO	PCV	SO
ELC							
Fear	0.111						
ILC	0.078	0.057					
JN	0.140	0.124	0.227				
MO	0.127	0.204	0.277	0.692			
PCV	0.130	0.067	0.259	0.584	0.536		
SO	0.096	0.150	0.275	0.639	0.708	0.661	

After fitting the measurement model, the structural model was run to test the hypotheses. H1 is statistically proved at a 1% significance level (β = 0.235, *p* = 0.002, *f*^*2*^ = 0.054, t = 3.07). The value zero didn’t lie in the confidence interval (0.097, 0.393). Except for this hypothesized relationship, other non-hypothesized direct relationships of MO and SO towards JN also proved with a 99% confidence interval. The q^2^, f^2^, and H^2^ for the model were found, and it was found that PCV has a medium-level effect size f^2^ (i.e., f^2^ > 0.02) and small predictive relevancy q^2^ (i.e., < 0.02) on JN, as the value of PCV on JN is less than 0.15. Overall, R² (i.e., 0.45) and the model explained 45% variation by this single exogenous construct on the endogenous variable, i.e., JN (See Table 5).

**Table 5:** Path significance and the structural relations related to basic model

H.no	Structural Path	Β	t-value	*P*-Value	R2	f^2^	Q^2^	Result
H1	PCV -> JN	(0.231)*	3.07	0.002	0.45	0.054	0.417	supported
Moderator	MO -> JN	(-0.372)*	5.31	0.00		0.141		**-**
Moderator	SO -> JN	(0.189)*	2.59	0.009		0.037		**-**
Marker	Fear -> JN	(-0.019)	-0.259	0.795		0		**-**
Control	ELC -> JN	(-0.037)	-0.659	0.51		0.002		**-**
Control	ILC -> JN	(-0.019)	-0.429	0.668		0		**-**
Control	Age -> JN	(-0.144)**	2.09	0.037				**-**
Control	Exp -> JN	-0.099	1.392	0.164				**-**

*, ** and *** represents 1%, 5%, 10% respectively. (based on two-tailed test)

### Interaction effect model

**Fig. 2:**
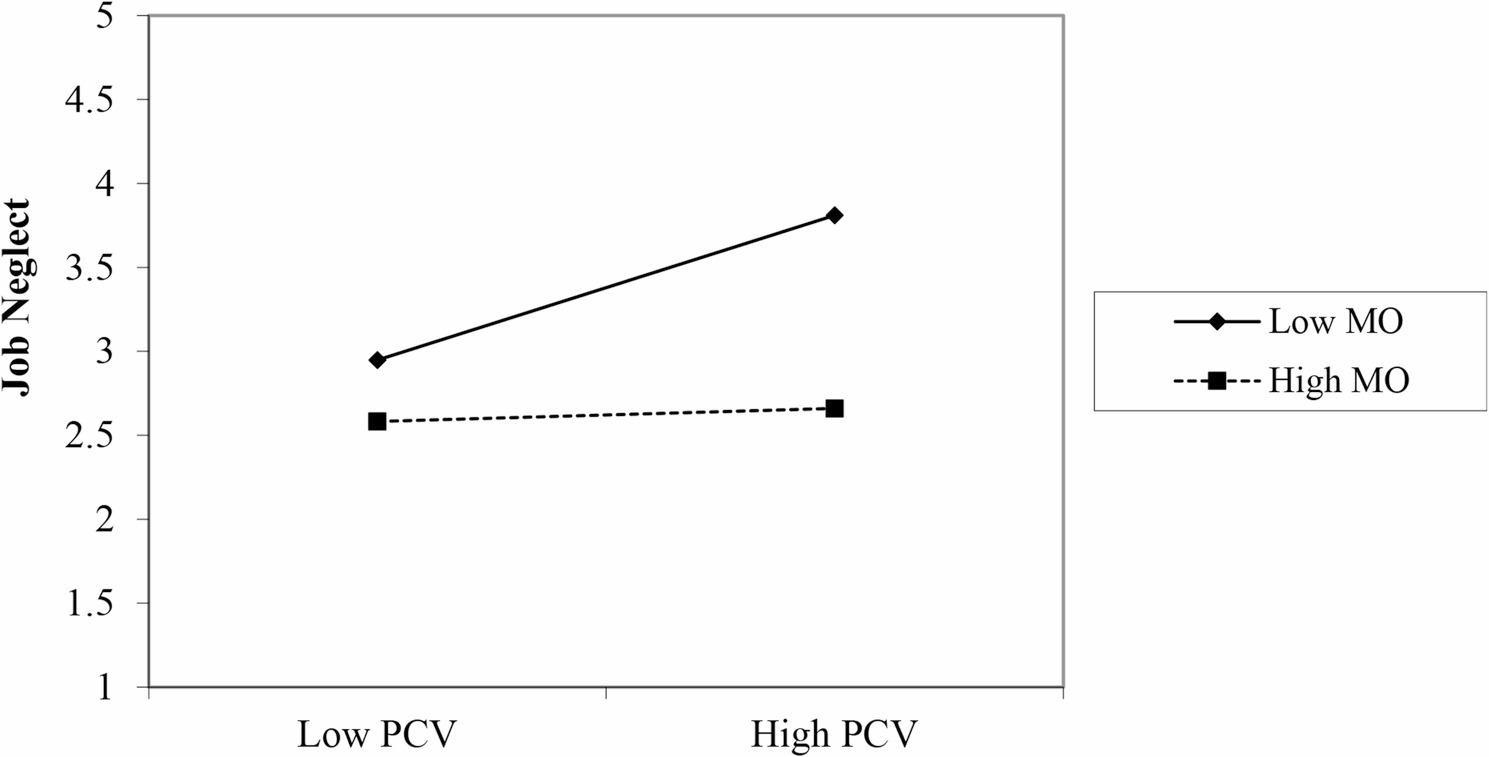
Two-way interaction between PCV and MO in the prediction of JN

Moderation has been tested by the product indicator approach, and it was found that H3 is proved with a 99% confidence interval (β = -0.221, *p* = 0.003, and t = 2.982) in which the zero does not fall (-0.361, -0.068), but it exerts a medium effect size on JN (i.e., f^2^ > 0.02) as suggested by [67]. That means high MO contributes positively to buffer the effects of PCV on JN, but H2 is not supported (β = -0.064, *p* = 0.447), as the t-values are below the threshold level (i.e., < 1.96). It means SO doesn’t have any significant impact between PCV and JN as a moderator. Zero was existing in the confidence interval (-0.237, 0,096). The MO relationship is also supported by the Jeremy Dowson moderation graph (see Fig. 2) [68]. It showed in a pictorial presentation that at a high level of MO, the line is relatively straighter than at a low stage of MO between the doctors working in public health care hospitals (See Table 6).

The fig. 2 above illustrate the graphical representation of the moderation effect of mastery orientation among psychological contract violation and job neglect.

**Table 6:** Path relation, values, f^2^ and significance status of interaction model

H #	Path Relation	β	Mean	St.Dev	t-values	*p*-values	Result
H2	(PCV*SO) -> JN	-0.064	-0.068	0.084	0.760	0.447	Not Supported
H3	(PCV*MO) -> JN	-0.221**	-0.216	0.074	2.982	0.003	Supported

*, ** and *** represents 1%, 5%, 10% respectively (based on two-tailed test)

### Multigroup moderation by gender

In the concluding phase of the study, we examined the significant differences between males and females regarding the impact of PCV on JN. We also examined the significant differences between males and females regarding the moderating impact of MO and SO between PCV and JN. So, H4 was significantly supported in the study. The differences in path coefficients indicated that the influence of PCV on the outcome i.e. JN was more pronounced in males than in females. On the contrary, the differences in path coefficients indicated that the moderating influence of both SO and MO in the impact of PCV on JN was stronger in females as compared to males. The findings of the multi-group analysis are presented in tables no. 7, 8, 9 and 10 below.

**Table 7:** Results of Differences (Male Vs Female)

	PLS-MGA		Parametric Test of Difference			Welch-Satterthwaite test		
Structural paths	Path Coefficients-diff (Public - Private)	*p*-Value original	Difference (Male - Female)	t value (|Male vs. Female|)	*p* value (Male vs. Female)	Difference (Male - Female)	t value (|Male vs. Female|)	*p* value (Male vs. Female)
ELC -> JN	0.088	0.535	0.088	0.656	0.512	0.088	0.608	0.544
Fear -> JN	0.178	0.42	0.178	0.994	0.321	0.178	0.921	0.359
ILC -> JN	-0.089	0.538	-0.089	0.616	0.539	-0.089	0.553	0.582
MO -> JN	-0.238	0.391	-0.238	1.024	0.307	-0.238	0.934	0.352
PCV -> JN	0.317	0.096	0.317	1.831	0.068	0.317	1.736	0.085
SO -> JN	0.528	0.07	0.528	1.79	0.075	0.528	1.61	0.11
MO x PCV -> JN	-0.184	0.216	-0.184	1.249	0.213	-0.184	1.229	0.222
SO x PCV -> JN	-0.193	0.234	-0.193	1.225	0.222	-0.193	1.207	0.230

**Table 8:** Bootstrapping results (Male Vs Female)

	Original (Female)	Original (Male)	Mean (Female)	Mean (Male)	STDEV (Female)	STDEV (Male)	t value (Female)	t value (Male)	*p* value (Female)	*p* value (Male)
ELC -> JN	0.013	0.102	-0.042	0.096	0.133	0.061	0.098	1.66	0.922	0.097
Fear -> JN	-0.13	0.048	-0.066	0.043	0.176	0.081	0.738	0.59	0.461	0.555
ILC -> JN	0.09	0.002	0.071	-0.003	0.155	0.045	0.584	0.04	0.559	0.968
MO -> JN	-0.16	-0.399	-0.049	-0.397	0.24	0.091	0.669	4.395	0.504	0.00
PCV -> JN	0.009	0.326	0.037	0.324	0.159	0.091	0.056	3.564	0.955	0.00
SO -> JN	-0.308	0.22	-0.032	0.217	0.315	0.095	0.976	2.314	0.329	0.021
MO x PCV -> JN	-0.003	-0.187	0.011	-0.171	0.119	0.092	0.029	2.033	0.977	0.042
SO x PCV -> JN	0.096	-0.097	-0.023	-0.097	0.126	0.099	0.762	0.977	0.446	0.328

**Table 9:** Results of multi-group analysis (Male Vs Female)

	PLS-MGA	
Structural paths	Path Coefficients-diff (Male - Female)	*p*-Value original
ELC -> JN	0.088	0.535
Fear -> JN	0.178	0.42
ILC -> JN	-0.089	0.538
MO -> JN	-0.238	0.391
PCV -> JN	0.317	0.096
SO -> JN	0.528	0.07
MO x PCV -> JN	-0.184	0.216
SO x PCV -> JN	-0.193	0.234

**Table 10:** Results of hypothesized relationships (Male Vs Female)

	Original (Female)	t value (Female)	*p* value (Female)	Result	Original (Male)	t value (Male)	*p* value (Male)	Result
ELC -> JN	0.013	0.098	0.922		0.102	1.66	0.097	
Fear -> JN	-0.13	0.738	0.461		0.048	0.59	0.555	
ILC -> JN	0.09	0.584	0.559		0.002	0.04	0.968	
MO -> JN	-0.16	0.669	0.504		-0.399	4.395	0.00	
PCV -> JN	0.009	0.056	0.955		0.326	3.564	0.00	Supported (Partially)
SO -> JN	-0.308	0.976	0.329		0.22	2.314	0.021	
MO x PCV -> JN	-0.003	0.029	0.977		-0.187	2.033	0.042	Supported (Partially)
SO x PCV -> JN	0.096	0.762	0.446		-0.097	0.977	0.328	

## Discussion

The results of this study largely align with previous research findings, which demonstrate that psychological contract violation (PCV) exerts a direct impact on deviant workplace behaviors, such as job neglect [34, 69, 70]. In the context of public healthcare in Pakistan, this relationship is particularly critical. Public hospitals cater to thousands of patients daily, many of whom cannot afford private healthcare and place high expectations on public services. Consequently, when doctors perceive that the organization has failed to fulfill its obligations, this violation may trigger neglect behaviors that could compromise patient care and the overall effectiveness of healthcare delivery [56].

The findings regarding mastery orientation (MO) as a moderator are consistent with prior literature [12, 71]. Individuals with high MO appear to interpret PCV within the broader organizational context, recognizing systemic constraints and the necessity of difficult decisions for the greater good. Such individuals tend to buffer negative emotional responses and exhibit lower engagement in counterproductive behaviors, consistent with Conservation of Resources Theory [44], which suggests that individuals strive to protect their psychological resources when faced with stressors. Another explanation to this relation can be grounded on Affective Events Theory presented by Weiss and Cropanzano [72], where people with high mastery orientation are expected to display minimal reaction in case where organization violates explicit or implicit contracts. Normally, such people consider these violations inevitable from the organizational point of view and do not consider them personal offense. Based on this logical reasoning, such employees tend to focus on the sustainable achievement of the organizational goals along with the provision of effective healthcare to the patients.

On the other hand, in this research study Subjugation Orientation has been tested to have an insignificant impact as a moderator among PCV and job neglect. The results are probably reflecting the professional characteristics of healthcare providers, where they are usually working in conditions in which they are solely responsible to take decisions in critical situations and carry the burden of responsibility as well. They are expected to demonstrate logical and critical solutions for the complex situations at hand. As Maznevski [43] explained in his study, these professionals may not exhibit the passive, externally oriented attributional style associated with SO. Abdullah et al. [73] in their study, proposed that the work nature of healthcare providers makes it mandatory to remain engaged in their work endeavor and come with decisions that are suitable with the prevailing situations, which in turn would control the chances of neglect behavioral outcomes in the prevalence of subjugation orientation.

Further, the current research executed the multi-group analysis where it was tested whether the gender plays the moderating role between PCV and job neglect. A significant impact of gender differences has been observed in the analysis. The results support the existing literature [74, 75], where it was suggested that, provided equal training opportunities and homogeneous work environment, the difference in the psychological responses of males and females can be controlled. Regardless of the gender, the healthcare providers are expected to demonstrate mastery and personal stimulus in their professional conduct, however, difference in the response to PCV is expected among different genders derived due to the physiological differences. An interesting insight has been observed in the study, where male doctors have been proved to be more demonstrate more compelling effect of job neglect in the presence of PCV. The finding explains a more sensitive approach of male doctors as compared to female doctors in the event of breach of psychological contract. On the other hand, female doctors may resort on other mechanisms to cope the stress or frustration of PCV, or may face the pressure to maintain their role in the presence of the expectations of the society that reduce the negative consequences of PCV [76, 77]. The results, therefore, emphasize the significance of keeping in view the impact of both situational and individual differences while evaluating workplace responses to psychological contract breaches.

To sum it up, the study findings strengthen the notion of including individual orientations and gender differences while studying the organizational violations of psychological contracts to enhance the theoretical and practical understanding of psychological contracts in healthcare settings.

### Theoretical and managerial contributions

The research findings, as discussed above, are in line with the previously conducted studies. Theoretically, the study was based on important theories; Affective Events Theory, Conservation of Resources Theory and Social Role Theory. The findings proved significant relationships among variables based on the theories embedded. The study, therefore, contributes theoretically by strengthening the established theories through the current set of study.

The role of healthcare providers, especially doctors, is crucial since human lives are dependent on their services. A major part on the population in developing countries, especially Pakistan, depend on public sector healthcare, given the affordability and financial constraints. Most of the people are unable to reach private sector healthcare and their last hope for a reasonable treatment resort on doctors of public sector hospitals. Since the predominant source of healthcare services rests with the public sector hospitals, the patients have high levels of expectations and hopes in receiving care and attention in public hospitals. Provided the crucial services expected from the doctors, job neglect can lead to disastrous outcomes even causing human casualties, which in any case is not acceptable.

The research focuses on the fact that even as a response to PCV, job neglect in healthcare profession is not an insignificant issue as it directly affects public health and patient safety. The results propose that when healthcare providers start neglecting their duties, it directly affects the standard of services provided, leading to potential harm to patients. In such cases, patients often question, “Whom to blame?” Therefore, to mitigate the adverse effects of PCV, governmental and healthcare management must take proactive steps to address both the causes and consequences of job neglect.

This study also provides new insights by demonstrating that the impact of PCV is moderated by Mastery Orientation (MO). High MO helps buffer the effects of PCV on job neglect, particularly in highly skilled professionals such as doctors. Administrative authorities need to understand the dynamics of individual interactions with the work environment and implement interventions that promote mastery-oriented thinking to reduce neglect behaviors. Understanding that doctors, with their high academic background, may interpret violations differently is essential for managers. The study emphasizes the need for the government and management to communicate to employees that certain breaches were necessary for survival and that they should view these violations in the long-term context of organizational improvement. The study signifies the moderating effect of Mastery Orientation (MO) that can reduce the impact of psychological contract violations on doctors’ job neglect, which indicates the need for managers and policymakers to introduce training, mentoring, and MO-aligned performance practices. It also highlights the importance of clear communication strategies among public sector doctors that openly explain unavoidable breaches in order to sustain commitment during organizational change.

Moreover, this study offers valuable insights for future researchers, particularly in the context of individual differences. The research on PCV is sparse in Pakistan, especially with regard to understanding how cultural and individual factors influence the perception of violations and job neglect. The model presented here also introduces the concept of cultural values and their impact on individual perceptions of workplace violations, offering a foundation for future studies in this area.

### Limitations and future directions

The current study offers a number of important contributions to both literature and practice, however, there are certain limitations which can be addressed in the upcoming studies in the same field. Firstly, the data has been collected in one shot which limits the potential to draw the study results over time, therefore, in future, longitudinal studies can be conducted. Cross sectional studies have limited generalizability, which is limits the scope of application of the research findings. The researchers must carry on longitudinal approach for data collection in future studies, to provide evidence of long-term impact of PCV on job neglect. It will enable us to enhance the understanding of the long-term impact of PCV on adverse employee outcomes. Moreover, the research has been conducted incorporating quantitative technique, where data has been collected from the respondents through questionnaires. In future, it is advised to conduct research using mixed-method approach, where qualitative data may be gathered from various sources as well. It will enable the researcher to get a holistic view of the phenomenon.

Furthermore, the study has taken individual cultural dimensions as moderators in the model. Particularly, mastery orientation and subjugation orientation have been tested in the research for their interplay between PCV and job neglect. It is recommended that in future research studies, other cultural dimensions may be explored to potentially pose moderating impact on the relationship. This inclusion will help understand how cultural values shape responses to PCV providing a more comprehensive view. In this study, only gender as a demographic moderator has been tested, whereas in future studies, the generational differences may also be studied where age groups will determine the intensity of relationship. In previous studies, demographic variable of age has been found to play a significant role on how employees cope with violations of psychological contracts [78]. It would be interesting to examine how age differences determine the probability of job neglect, especially in relation to different stages of career development and psychological resilience.

Another area for future research can be examining the impact of PCV on employee well-being among different organizational contexts. The studies may incorporate both quantitative and qualitative methods, where researchers could gain deeper insights into how PCV leads to emotional and physical stress, particularly in high-stakes professions such as healthcare. Longitudinal research could also assess the post-violation outcomes, investigating how the long-term consequences of PCV manifest in different sectors.

## Conclusion

To summarize the research and findings, it is concluded that it is imperative for academic researchers and practitioners to focus on the multifaceted issue of job neglect, especially in the sensitive sector of healthcare. Moreover, PCV has been tested and proved to be a major cause of job neglect among doctors. Such adverse behaviors result in the overall deterioration of the organizational performance and service receivers’ wellbeing. The study further explains the interplay of mastery orientation, along with the individual impact of PCV, where organizations can reduce the impact of job neglect by incorporating the findings properly. The research highlights the significance of providing environment, where employees or doctors can maintain the sense of agency and responsibility, even when faced with organizational challenges.

Moreover, the results also underscore the demand for a profound understanding of how cultural values—such as MO and SO affect employees’ responses to violations of psychological contracts. These insights not only contribute to the theoretical understanding of PCV but also provide practical guidance for healthcare organizations aiming to minimize job neglect. Management must focus on communication and support systems that align organizational goals with employees’ values, ensuring that breaches are viewed as necessary steps for future growth rather than as personal violations.

## Appendix

**Fig. 3:**
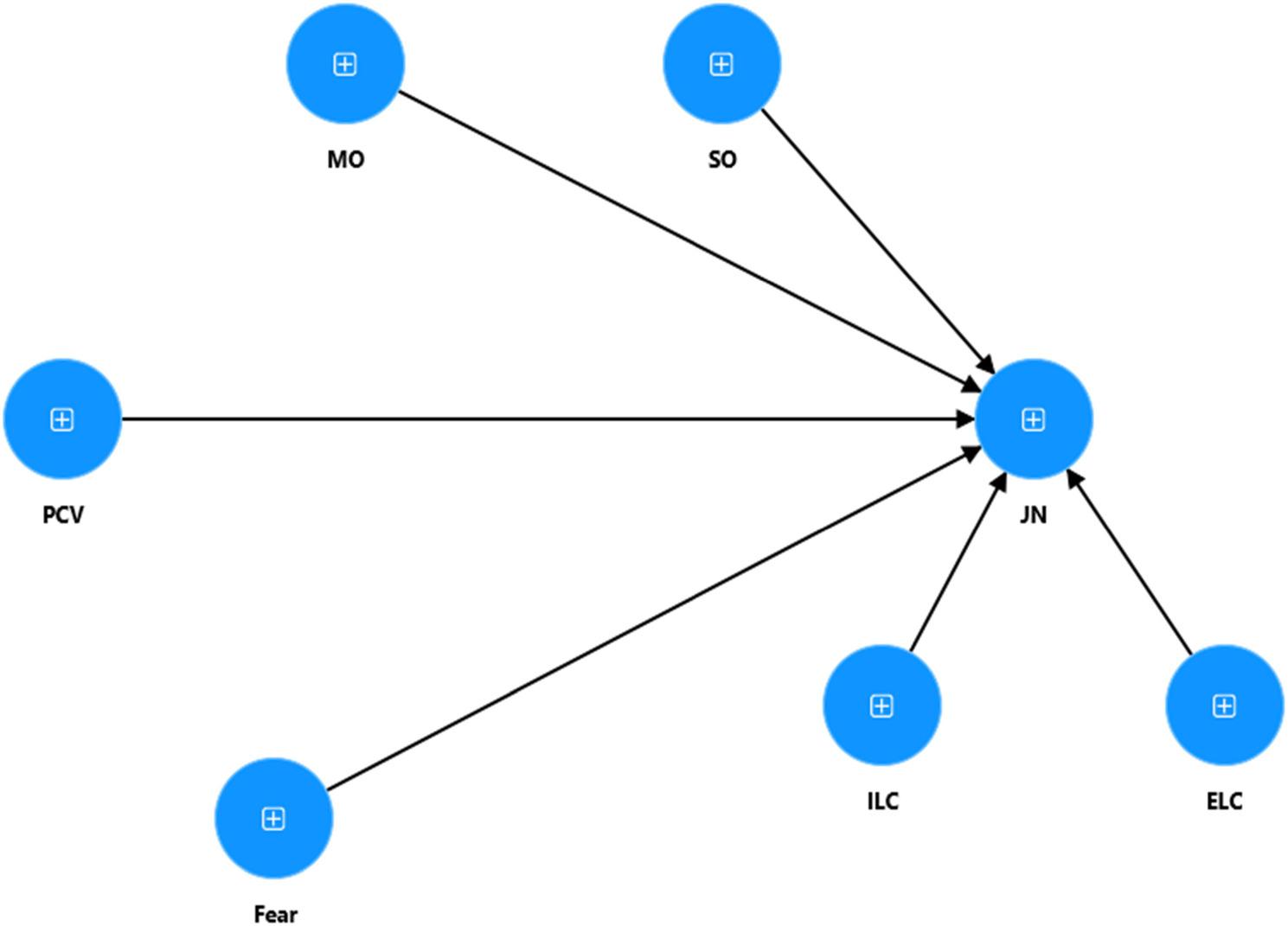
Measurement model

**Fig. 4:**
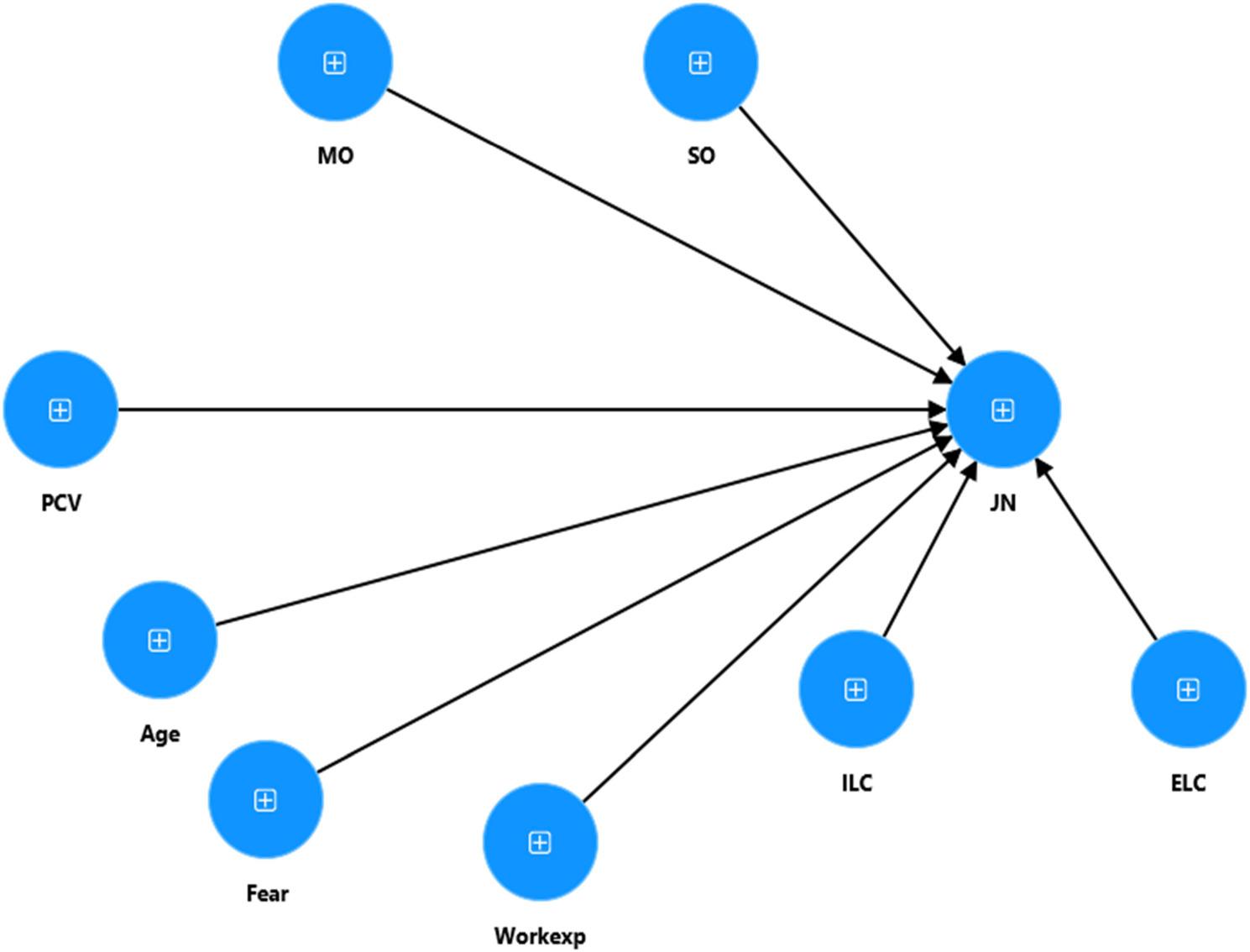
Structural model

**Fig. 5:**
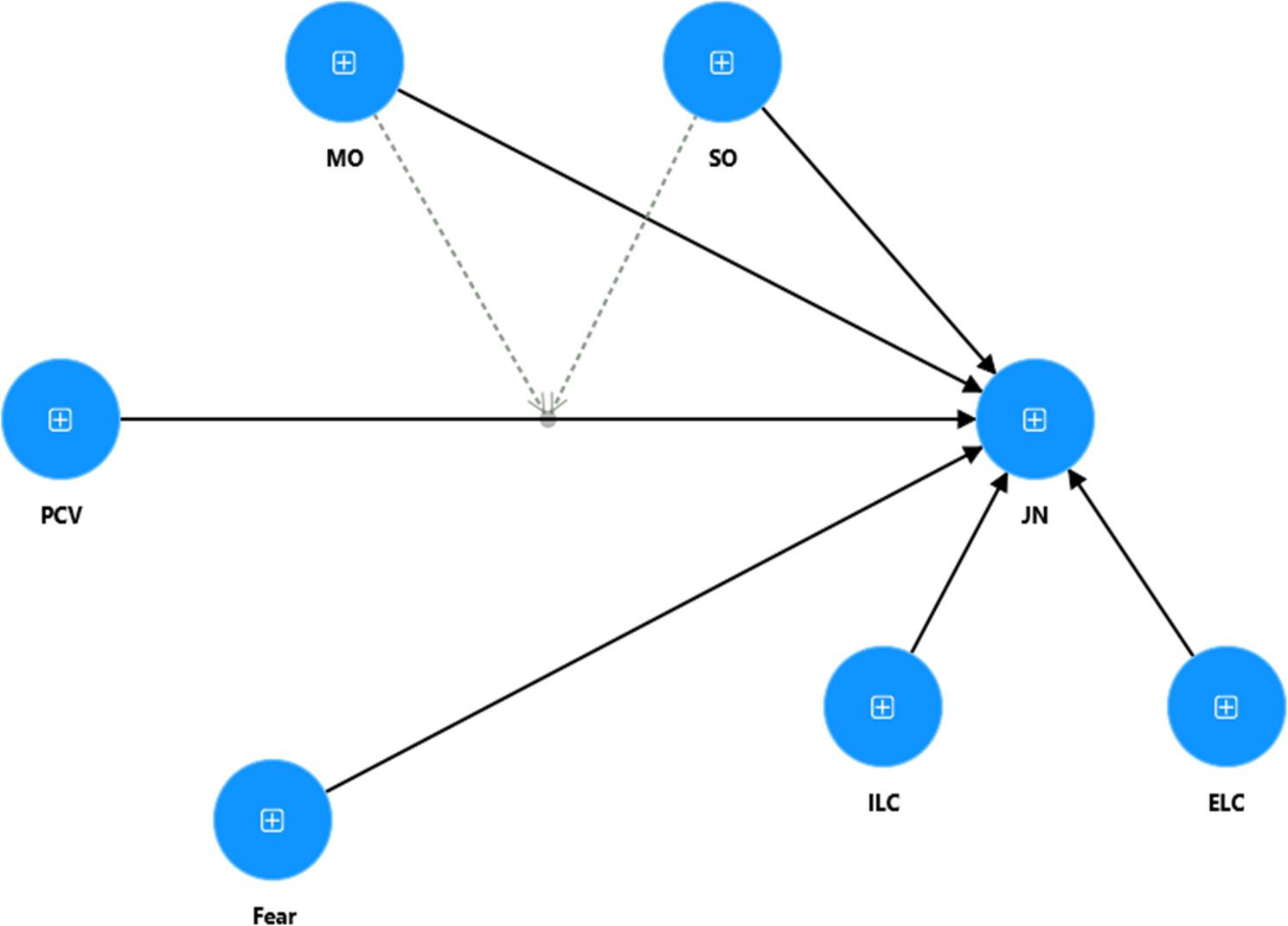
Moderation model

## Data Availability

Data will be made available on reasonable request from the corresponding author.

## Abbreviations

PCV: Psychological Contract Violation
PCB: Psychological Contract Breach
COR: Conservation of Resources Theory
AET: Affective Event Theory
SET: Social Exchange Theory
JN: Job Neglect
SO: Subjugation Orientation
MO: Mastery Orientation
